# Gut Microbiomes of Freshwater Mussels (Unionidae) Are Taxonomically and Phylogenetically Variable across Years but Remain Functionally Stable

**DOI:** 10.3390/microorganisms9020411

**Published:** 2021-02-16

**Authors:** Mark McCauley, Marlène Chiarello, Carla L. Atkinson, Colin R. Jackson

**Affiliations:** 1 Department of Biology, University of Mississippi, University, MS 38677, USA; marlene.chiarello@gmail.com (M.C.); cjackson@olemiss.edu (C.R.J.); 2 Department of Biological Sciences, University of Alabama, Tuscaloosa, AL 35401, USA; carla.l.atkinson@ua.edu

**Keywords:** bivalve, host–microbe interactions, predicted metagenome function, 16S rRNA

## Abstract

Freshwater mussels perform essential ecosystem functions, yet we have no information on how their microbiomes fluctuate over time. In this study, we examined temporal variation in the microbiome of six mussel species (*Lampsilis ornata*, *Obovaria unicolor*, *Elliptio arca*, *Fusconaia cerina*, *Cyclonaias asperata*, and *Tritogonia verrucosa*) sampled from the same river in 2016 and 2019. We examined the taxonomic, phylogenetic, and inferred functional (from 16S rRNA sequences) facets of their microbiome diversity. Significant differences between the two years were identified in five of the six species sampled. However, not all species that exhibited a temporally variable microbiome were functionally distinct across years, indicating functional redundancy within the mussel gut microbiome. Inferred biosynthesis pathways showed temporal variation in pathways involved in degradation, while pathways involved in cellular metabolism were stable. There was no evidence for phylosymbiosis across any facet of microbiome biodiversity. These results indicate that temporal variation is an important factor in the assembly of the gut microbiomes of freshwater mussels and provides further support that the mussel gut microbiome is involved in host development and activity.

## 1. Introduction

Freshwater mussels (Family Unionidae) are a diverse (~300 species in North America) but highly imperiled fauna group, with the majority of species located in the Southeastern United States [1]. Historically, while freshwater mussels were highly abundant and ubiquitous across North America, approximately 65% of freshwater mussel species are threatened or endangered, primarily as a result of anthropogenic disturbances [2,3,4]. With projections of further losses in freshwater mussel biodiversity [5], there will likely be significant declines in abundances and a reduction in the ecosystem services that they provide [6].

Mussel holobionts play an essential role in aquatic ecosystems by performing numerous important functions, including filter feeding, excreting soluble nutrients, and biodepositing organic material [6,7], during their lifespan of up to 100 years [8]. Functionally, these processes contribute to critical ecosystem services such as supporting food webs, biofiltration, nutrient cycling, nutrient storage, and improving water quality [6]. In invertebrates, the bacterial microbiome interacts with numerous host and ecosystem functional processes, as described in sponges and corals [9,10]. However, in freshwater mussels, despite recent evidence suggesting that the gut microbiome plays a role in nutrient digestion [11,12], data on how the microbiome varies across species and environmental conditions, and the functional role of the microbiome within mussel holobionts is still largely unexplored [12].

The gut microbiome of freshwater mussels is distinct from the water column, species specific, and spatially dependent [13]. While phylosymbiosis, a correlation between host phylogeny and microbiome composition, was not detected on seven species from the Unionidae family (*Amblema plicata*, *Obliquaria reflexa*, and *Pleurobema decisum*, in addition to the six included in this study), there were significant phylogenetic patterns in the structure of the microbiome [12]. However, prior studies on the microbiomes of freshwater mussels were based on data from one season of sampling [13,14,15], and the microbiome of marine bivalves has been shown to vary temporally [16,17,18]. It is unknown how the gut microbiomes of freshwater mussels may vary from year to year, which could be substantial given their long lifespans.

Here we examined the gut microbiome of six species of freshwater mussels spanning three distinct phylogenetic tribes (Lampsilini, Pleurobemini, and Quadrulini) that have been previously shown to occupy different functional niches in that they require different elements for maintenance and growth and excrete nutrients at varying ratios [19]. Thus, the aims of this study were (1) to investigate the structure of gut microbiomes of freshwater mussels sampled over different years, (2) to correlate temporal changes in microbiome structure to their inferred functionality, and (3) determine if this varied as a response of species or tribe identity.

## 2. Materials and Methods

### 2.1. Sampling

**Figure 1:**
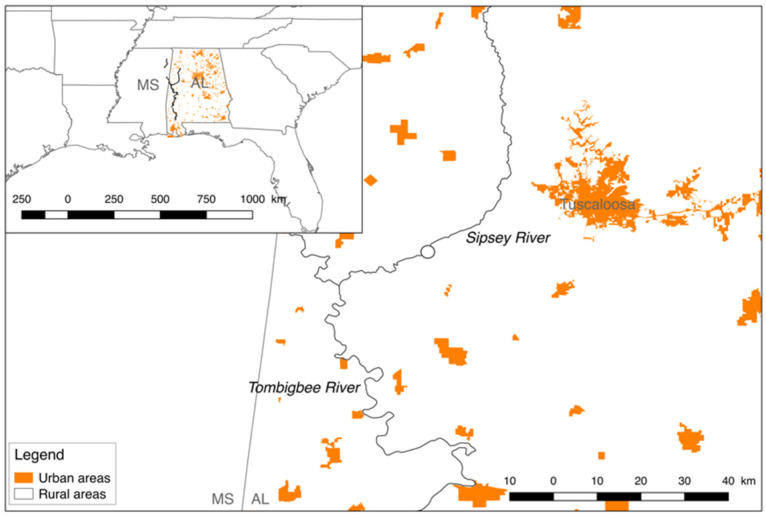
Six freshwater mussel species were collected between August 2016 and July 2019 from a site (circled) on the Sipsey River, Alabama, USA. The Sipsey River is surrounded by rural lands (white), with the city of Tuscaloosa representing the closest and largest urban area (orange). Temperature, pH, dissolved oxygen (mg/L), and ammonia, orthophosphate, phosphorus, nitrate, and nitrite of the water column were collected. The river flow is from north to southwest.

Six mussel species (*Cyclonaias asperata*, *Elliptio arca*, *Fusconaia cerina*, *Lampsilis ornata*, *Obovaria unicolor*, and *Tritogonia verrucosa*) from three tribes (Lampsilini, Pleurobemini, and Quadrulini) were collected from the same site on the Sipsey River (Alabama, US) between August 2016 and July 2019 (Figure 1). In 2016, the individuals were transported alive and covered in moist towels in coolers to the University of Alabama, and in 2019, they were placed on ice for transportation back to the University of Alabama. For each species, 4–5 individuals were collected in August 2016, and 9–10 individuals were collected in July 2019 (Supplementary Table S1). Physicochemical parameters, including temperature, pH, and dissolved oxygen (mg L^−1^), were measured using a calibrated multiparameter sonde (YSI Inc., Yellow Springs, OH) in both years. Water samples were collected, filtered (47 mm GF/F; 0.7 μm pore size; EMD Millipore, Buckinghamshire, UK), and analyzed for ammonia (μg L^−1^), orthophosphate (μg L^−1^), phosphorous (μg L^−1^), nitrate (μg L^−1^), and nitrite (μg L^−1^) using a Lachat QuikChem FIA +8000 Series flow injection analyzer (Hach Company, Loveland, CO, USA) (Supplementary Table S2). At each site, we determined the mean channel width and measured stream discharge with an FH950 flow meter (Hach, Loveland, CO). Mussels were collected in 2016 under the authority of the USFWS (Permit No.: TE68616B-1) and ALCDNR (Permit No.: 2016077745468680) permits, in addition to the ALCDNR (Permit No.: 2019118497068680) permit in 2019. The majority of the Sipsey River is considered relatively pristine given the lack of anthropogenic disturbance, and it supports a diverse assemblage of aquatic species [19,20]. No major disturbance events were recorded between our sampling points. Mussel gut tissues were standardized by weight (~0.8 g), extracted with sterile dissecting equipment, and stored at −80 °C before being transported to the University of Mississippi. Mussel shells were measured each year and their ages inferred (using regressions published in [9]) and compared using a Student’s *t*-test with unequal sample sizes.

### 2.2. DNA Extraction: 16S rRNA Gene Sequencing

Gut tissue was ground within an extraction buffer from a Qiagen DNeasy PowerSoil Pro kit (Qiagen, Germantown, MD) using Pellet Pestles (Fisher, Pittsburgh). The homogenate was then briefly vortexed, centrifuged (2000× *g*, 1 min), and ground again before a second centrifugation step to the pellet host tissue. The supernatant containing bacteria was eluted and used in the standard extraction protocol. Dual-indexed barcoded primers were used to amplify the V4 region of the 16S rRNA gene of the extracted DNA following established techniques [21]. The amplified 16S rRNA gene fragments were combined and spiked with 20% PhiX before being sequenced on an Illumina MiSeq at the University of Mississippi Medical Center Molecular and Genomics Core facility.

### 2.3. Sequence Processing

Raw sequence files (FASTQ) from the 59 individuals sampled in 2019 were combined with 29 FASTQ sequences previously reported [12,13] of the same species sampled from the same site in 2016 (Supplementary Table S1). Together, these sequences were processed in mothur v. 1.41.1 [22] using the pipeline and mothur SOP (https://www.mothur.org/wiki/MiSeq_SOP (accessed on 23 January 2021)) referenced in July 2020. Sequences were aligned to the Silva database release 132 [23] and classified based on RDP version 16 [24]. After the removal of nonbacterial sequences, six individuals were excluded from further analysis because they contained <1000 sequences, with 82 mussel microbiome samples comprising the final dataset. We then grouped sequences into operational taxonomic units (OTUs) based on 97% identity and randomly subsampled 1000 sequences from each individual to correct for uneven sequencing efficiency. A total of 7196 singleton OTUs (represented by just one sequence) were removed, leaving 15,751 OTUs for further analysis. After rarefaction, coverage averaged 0.91 ± 0.08 across all individuals.

### 2.4. Construction of Bacterial and Host Phylogenetic Trees

A bacterial phylogenetic tree grouping all OTUs was obtained by inserting their reference sequence into the Greengenes 99% OTUs reference phylogenetic tree [25] version 13.8 using the *SEPP* insertion tool [26] provided in QIIME2 v. 2019-4 [27] with default parameters.

A mussel phylogenetic tree (Family Unionidae, subfamily Ambleminae) was created with sequences for two nuclear markers (ITS 1 and 5.8S rRNA) and three mitochondrial markers (CO1, NADH, and 16S rRNA) that were downloaded from GenBank (Supplementary Table S3) and aligned (default parameters) using Geneious 9.1.6 (http://www.geneious.com (accessed on 23 January 2021)). Consensus gene sequences were concatenated with the Muscle algorithm [28] using the default parameters within Geneious (v. R10.2). The most appropriate substitution model was selected with Partitionfinder 2 [29]. A Monte Carlo run of four independent Markov chains, with 10 million generations each sampling every 1000 generations, was conducted utilizing the MrBayes plugin for Geneious [30].

### 2.5. Microbiome Analysis

Alpha diversity within each microbiome was calculated by using three complementary indices, namely OTU richness (observed number of OTUs, Sobs), the Shannon index, and the Inverse Simpson index using *vegan* and compared with ANOVA. Pairwise phylogenetic dissimilarities between bacterial communities were assessed using Weighted Unifrac (W-Unifrac) in the R package *GUniFrac* and bacterial phylogeny [31]. Bray–Curtis dissimilarities were calculated using the “vegdist” function provided in the *vegan* R package [32]. Differences in microbiome structure between mussel species, collected in the same year, were assessed using PERMANOVA on both W-Unifrac and Bray–Curtis dissimilarities using the “adonis” function provided in *vegan* (999 permutations) and associated post-hoc pairwise comparisons using the “pairwise.adonis” function (https://github.com/pmartinezarbizu/pairwiseAdonis (accessed on 23 January 2021)). Homogeneity of dispersion was calculated using the function “permutest.betadisper” provided in *vegan*. Nonrepeated measures PERMANOVAs were conducted on mussel species that were collected between 2016 and 2019 with the packages *BiodiversityR* [33] and *vegan*.

Phylosymbiosis (correlation between the mussel phylogeny and microbiome dissimilarities) was tested using both Bray–Curtis and W-Unifrac distance matrices [12]. Mantel tests between branch lengths on mussel phylogenetic tree and Bray–Curtis and W-Unifrac values computed on species-average microbiomes were computed using *vegan* (Spearman’s coefficient, 999 permutations). Complementarily, microbial dendrograms representing Bray–Curtis and W-Unifrac values computed on species-average microbiomes were created using a hierarchical cluster analysis using the “hclust” function from the *stats* R package. Cophyloplots representing such dendrograms and mussel phylogeny were visualized using *ape*. Disagreement between their topologies was then assessed using the unweighted Robinson–Foulds distance, sensitive to tree topology only, using the “treedist” function provided in the *phangorn* R package [34]. Correlation between branch lengths was assessed using Spearman’s correlation using the “cor_cophenetic” functions from the *dendextend* R package [35].

Co-occurrence analysis of bacterial OTUs was conducted on both 2016 and 2019 timepoints utilizing the “co-occurrence_network” function of the *microbiomeSeq* package [36]. Strong correlations (ρ > 0.75, *p* < 0.05) between bacterial genera were identified for each year, with the correlation matrix exported to, and analyzed with, Cytoscape version 3.8.0 [37]. Finally, species core microbiomes were defined as OTUs present in at least 95% of individuals of a given species during each year, with a minimum relative abundance of 0.1%.

### 2.6. Inference of Bacterial Functions

Estimation of the potential microbiome functionality based on 16S rRNA data was conducted by comparing software reference OTU sequences to referenced genomes in the MetaCyc database version 2019 [38] using PICRUST2 [39] with default parameters. The resulting average nearest sequenced taxon index was 0.12 ± 0.03 across all samples. Using the *Deseq2* package [40] differential expression analyses were conducted to detect significant log-fold changes between the predicted relative abundances of inferred pathways across time points, using an adjusted *p*-value of 0.05. Relative abundances of averaged functional pathways within species were then transformed into Bray–Curtis dissimilarities and tested for phylosymbiosis using a Mantel test with host phylogenetic distances.

## 3. Results

### 3.1. Composition of Unionidae Gut Microbiomes

**Figure 2:**
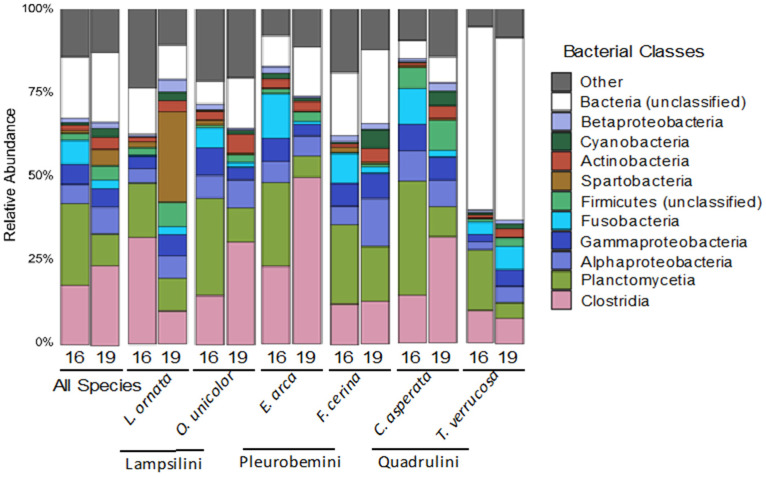
Relative abundance of the ten most abundant bacterial phyla/classes in the gut microbiome of six species of Unionidae mussels sampled between 2016 and 2019 at the same site on the Sipsey River, AL, USA, as determined from 16S rRNA gene sequence counts. Mussel species were *Lampsilis ornata*, *Obovaria unicolor*, *Elliptio arca*, *Fusconaia cerina*, *Cyclonaias asperata*, and *Tritogonia verrucosa*. Tribes are listed below member species. Each bar represents the average microbiome for species collected in 2016 (16) and 2019 (19). Class “Other” includes 74 bacterial classes, each less than 2% of sequences in the entire dataset.

Overall, the relative abundances of bacterial phyla present in freshwater mussel microbiomes sampled in 2016 were similar to those sampled in 2019, with three phyla accounting for 58.6% of all sequence reads in 2016 and 54.3% in 2019. These were identified as Firmicutes (21.5% and 26.7% for 2016 and 2019, respectively), Proteobacteria (12.9% and 15.2%, respectively), and Planctomycetes (which significantly declined from 24.2% in 2016 to 12.4% in 2019). Proteobacteria consisted mostly of Gammaproteobacteria (5.9% and 5.4% of all sequences in 2016 and 2019, respectively) and Alphaproteobacteria (5.6% and 7.0%, respectively), Firmicutes consisted mostly of Clostridia (18.5% and 22.6%, respectively), and Planctomycetes consisted solely of Planctomycetia. Outside of Proteobacteria and Firmicutes, the most abundant classes were Cyanobacteria (7.1% of all reads in both years) and Fusobacteriia, which significantly declined from 6.9% in 2016 to 2.5% in 2019 (Figure 2). Additionally, 18.1% and 20.6% remained unclassified at the phylum level in 2016 and 2019, respectively (Figure 2). The number of unclassified sequences at the phylum level was species dependent (Kruskal–Wallis, *p* < 0.05), with *T. verrucosa* containing the highest percentage in both years (54.6% in 2016 and 54.0% in 2019).

In 2019, nine OTUs represented approximately 50% of the data, each accounting for 3.4-10.6% of the total reads. OTUs 1, 2, and 3 were classified as members of the order Clostridiales (Firmicutes), OTU 4 was identified as a Rhizobiales (Proteobacteria), OTU 5 was an unclassified bacterium, and both OTUs 6 and 9 were classified as Planctomycetales (Planctomycetes). OTU 7 was classified within the order Fusobacteriales (Fusobacteriia) and OTU 8 within the class Spartobacteria. OTUs 1-6 and 9 had a similar relative abundance in 2016 and 2019, while OTUs 7 and 8 were more than twice as abundant in 2019 than in 2016.

### 3.2. Temporal Variation in Unionidae Gut Microbiomes

**Figure 3:**
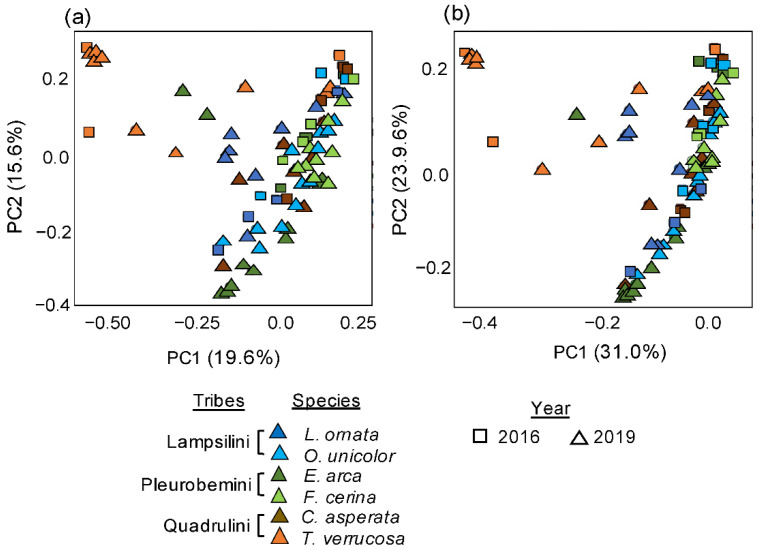
Principle coordinates analyses representing Bray-Curtis (**a**) and W-Unifrac dissimilarities (**b**) between six species of Unionidae mussels sampled at the same site on the Sipsey River, AL, USA, across two years. Mussel species were *Lampsilis ornata*, *Obovaria unicolor*, *Elliptio arca*, *Fusconaia cerina*, *Cyclonaias asperata*, and *Tritogonia verrucosa*. The overall effects of species and year on microbiome structure were assessed using separated PERMANOVAs, with results (*p* < 0.001 for all) indicated on each plot.

Overall, there was a moderate but significant temporal difference between microbiomes sampled in 2016 and 2019 (PERMANOVAs on Bray–Curtis, R^2^ = 0.08 *p* < 0.001; W-Unifrac, R^2^ = 0.09, *p* < 0.001; Figure 3a,b) alongside a stronger species effect (PERMANOVAs on Bray–Curtis and W-Unifrac, *p* < 0.001, R^2^ = 0.23 and 0.25, respectively, Figure 3a,b). The “temporal*species” interaction factor was also significant (PERMANOVAs on Bray–Curtis and W-Unifrac, *p* < 0.05, R^2^ = 0.07 and 0.09). Post-hoc pairwise comparisons revealed that all species except *T. verrucosa* (Bray–Curtis and W-Unifrac, *p* > 0.05 for both) had a significantly different microbiome across years (*p* < 0.05) (Table 1). No significant differences in variability of microbiome structure within species were detected between years. Further, no differences were detected between species alpha diversity indices between 2016 and 2019 (Table 1, Supplementary Figure S1).

Given that individuals collected from each mussel species in 2016 and 2019 were not significantly different in length (*p* > 0.1), we estimated similar ages of the individuals during both samplings (Supplementary Table S1). Site physicochemistry was not significantly different between years (*p* > 0.05, Supplementary Table S2).

### 3.3. Testing for Phylosymbiosis in Unionidae Gut Microbiomes

While distinct microbiomes were detected across individuals belonging to the three distinct phylogenetic tribes (PERMANOVAs on Bray–Curtis and W-Unifrac, *p* < 0.01, R^2^ = 0.12 and 0.14, respectively), there was no phylosymbiosis pattern detected between mussel phylogenetic relatedness and microbiome dissimilarities (Mantel tests based on Spearman’s coefficient, *p* > 0.05, R^2^ = 0.1–0.4 for Bray–Curtis and W-Unifrac dissimilarities, respectively). Further, there was no significant correlation between the structure of the mussel phylogeny and microbiome dendrograms (Robinson–Foulds distance of 1.0, and cophenetic correlation, *p* > 0.05 for all Bray–Curtis and W-Unifrac comparisons).

### 3.4. Temporal Variation in Unionidae Gut Core Microbiomes

Species core microbiomes showed temporal variation with 33% of all of core OTUs (OTUs present in >95% individuals in a given year), being present in fewer than 50% of individuals during the other year. The relative abundance of core OTUs that were specific to one year was tribe dependent (*p* < 0.05), with a mean (±SE) variability of 44.2 ± 4.3% in *L. ornata* and *O. unicolor* (Lampsilini), 30.1 ± 1.6% in *E. arca* and *F. cerina* (Pleurobemini), and 24.5 ± 3.8% in *C. asperata* and *T. verrucosa* (Quadrulini). In 2019, four of the six species (*C. asperata*, *L. ornata*, *O. unicolor,* and *T. verrucosa*) contained a core OTU, OTU 16, which was classified within the order *Synechococcus* (Cyanobacteria), and present in very few individuals in 2016 (0.77% relative abundance in 2019 compared to 0.21% in 2016). Three species (*E. arca, F. cerina*, and *O. unicolor*) hosted two novel core OTUs that were much less represented in 2016, OTU 17 (0.82% in 2019 compared to 0.05% in 2016), and OTU 23 (0.79% in 2019 compared to 0.18% in 2016), both of which classified as a *Mycobacterium* (Actinobacteria).

Network analysis detected that none of the strong (>0.75 rho) significant correlations between bacterial phyla identified in 2016 were still present in 2019 (Supplementary Table S4). While the same bacteria often appeared in the same host species across years, the patterns of co-occurrence shared between bacteria were not temporally stable (Supplementary Table S4). Across all species characterized in 2016, Spirochaetes and BRC1, alongside Armatimonadetes and Deinococcus-Thermus were significantly correlated, whereas in 2019, Armatimonadetes significantly associated with Ignavibacteriae and Proteobacteria was strongly associated with Planctomycetes (Supplementary Table S2).

### 3.5. Temporal Variation in the Inferred Microbial Functionality

**Figure 4:**
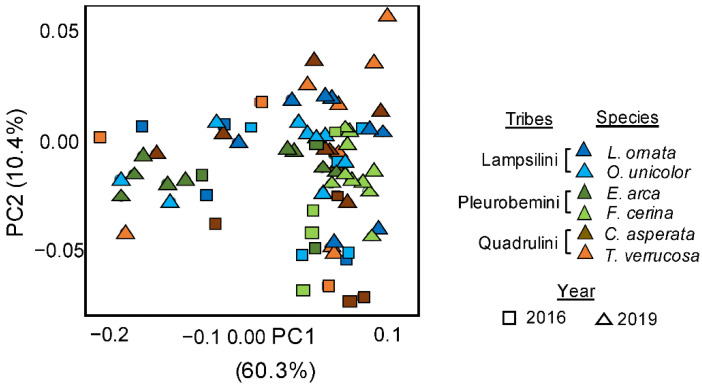
Principal co-ordinates analyses representing Bray–Curtis dissimilarities calculated from inferred functional pathways from 16S rRNA gene data between six species of Unionidae mussels sampled at the same site on the Sipsey River, AL, USA, across two years. Mussel species were *Lampsilis ornata*, *Obovaria unicolor*, *Elliptio arca*, *Fusconaia cerina*, *Cyclonaias asperata*, and *Tritogonia verrucosa*. The overall effects of species and year on microbiome structure were assessed using separated PERMANOVAs, with results (*p* < 0.001 for all) indicated on each plot.

There was a significant temporal effect between inferred microbial functionality inferred in 2016 and in 2019 (PERMANOVAs on Bray–Curtis, R^2^ = 0.12 *p* < 0.001, Figure 4), alongside a significant and stronger species effect (PERMANOVAs on Bray–Curtis and W-Unifrac, *p* < 0.001, R^2^ = 0.29, Figure 4), and a significant temporal*species interaction (PERMANOVAs on Bray–Curtis and W-Unifrac, *p* < 0.05, R^2^ = 0.11). However, post-hoc pairwise comparisons found that only differences between two species, *L. ornata* (adjusted *p* = 0.027) and *F. cerina* (adjusted *p* = 0.003) drove this temporal significance, with the inferred microbial functionality of the four remaining species similar between both years (Table 1). No signal for phylosymbiosis was detected based on either the 2016 or 2019 functional data (Mantel tests based on Spearman’s coefficient; Bray–Curtis *p* > 0.05).

**Figure 5:**
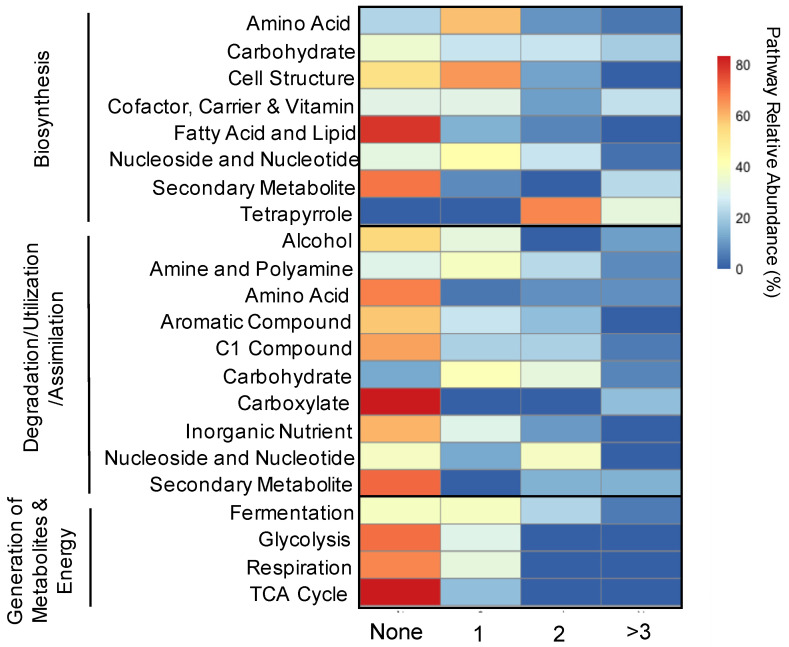
Relative abundances of inferred functional pathways between microbiomes of six species of Unionidae mussels sampled at the same site on the Sipsey River, AL, USA, across two years. Pathways that were not significantly different (adjusted *p* < 0.05) in any species (None) are presented alongside pathways that were found to be significantly different in one (1), two (2), and at least three (<3) species sampled in 2019 compared to 2016. Color denotes the percentage of inferred pathways. Parent functional pathways are labeled from the MetaCyc database version 2019. Mussel species were *Lampsilis ornata*, *Obovaria unicolor*, *Elliptio arca*, *Fusconaia cerina*, *Cyclonaias asperata*, and *Tritogonia verrucosa*.

Of the three major superclasses of pathways denoted by MetaCyc, more pathways within the biosynthesis superclass (mean = 63.1 ± 25.5%) differed between years, with significant positive and negative log-fold changes in the predicted relative abundances for at least one mussel species than pathways within the degradation/utilization/assimilation superclass (mean = 46.4 ± 22.8%) or in the generation of precursor metabolites and energy superclass (26.1 ± 21.2%) (Figure 5). Further, within the biosynthesis pathway superclass, pathways involved in the biosynthesis of carbohydrates, cofactors and vitamins, and tetrapyrrole all had between 70 and 100% of pathways significantly different between years in at least one mussel species and from 20.0 to 33.3% significantly different in at least three species (Figure 5). The most stable pathway within the biosynthesis pathway superclass was involved with cellular structure, with only 21.4% variation across one species and 0% across at least three (Figure 5). Three parent pathways within the degradation/utilization/assimilation superclass, involving the degradation of carbohydrates, carboxylates, and nucleosides, were 62.5–86.7% variable between years in at least one species but less than 6.6% variable in at least three species (Figure 5). The other five main parent pathways had less than 41.7% variability in one species and less than 16.7% in at least three (Figure 5). Three of the four pathways within the generation or precursor metabolites and energy superclass, Glycolysis, TCA Cycle and Respiration, were more conserved, with less than 30% variation in one mussel species and 0% in at least two species.

## 4. Discussion

By investigating the gut microbiomes of six freshwater mussel species sampled from the same site during the summers of 2016 and 2019, we detected significant temporal variation in the taxonomic and phylogenetic structure of the bacterial community. However, just two of the five mussel species showing temporal variability also exhibited significant variation in the inferred bacterial functionality between the two years. While previous studies have identified significant interspecies variation in the microbiomes of freshwater mussel species [12,13], between those sampled in the wild and in captivity [15], this is the first report of year-to-year variation in the microbiome within freshwater mussels. Temporal analysis of wild animal microbiomes are uncommon, and this represents only the second that documents microbial variation in wild bivalves (16).

Across the two years of summer sampling, temporal variation explained a smaller percentage of the taxonomic (8%) and phylogenetic (9%) structure of the microbiome when compared to host species (23% and 25%, respectively) and spatial variation [12] between two sites 30 km apart in 2016 (14% and 17% respectively). This suggests a greater species and spatial influence on microbiome diversity than temporal changes. Similar findings have been reported in other aquatic invertebrates, with significant environmental and temporal influences on the structure of microbiomes associated with the Eastern Oyster (*Crassostrea virginica*) [16,17], the Pacific oyster (*Crassostrea gigas*) [18,19], and the European lobster (*Homarus gammarus*) [41]. Given the significant variation in annual discharge, dissolved inorganic carbon, and nitrate nitrogen throughout the Sipsey River from 2015 to 2017, including our sample site [19], it is likely that these changing environmental factors over time contributed to the microbial variation recorded in our study.

While there was structural variation in the microbiomes of five out of the six sampled mussel species, this did not result in changes in inferred functionality. This suggests a degree of functional redundancy present in the microbiome of these mussel species. While not previously reported in bivalves, functional redundancy has been identified in another mollusk, the River Nerite (*Theodocus fluviatilis*) [42]. Of the three mussel species that exhibited functional redundancy (*O. unicolor*, *E. arca*, and *C. asperata*), each had significant year-to-year variation in the relative abundances of three abundant bacterial families (Fusobacteriaceae, Planctomycetaceae, and Clostridiaceae). Each of these families was also abundant in previously published microbiomes that also exhibited functional redundancy [43,44,45]. The apparent functional redundancy may result in increased functional stability and resilience against not only naturally fluctuating conditions but also anthropogenic disturbances [46]. While we predicted good accuracy of our inferred functions (NSTI distances: 0.12 ± 0.03), caution must be applied when inferring bacterial functionality, as predictions may not reveal specialized functions that are expressed at the transcriptional level [47]. As many of the current inferred pathways cannot be fully measured, continued investigation utilizing metatranscriptomic data is therefore required for further assessment of the functional variation of the mussel microbiome when facing changing conditions.

Biosynthesis pathways were significantly more variable between August 2016 and July 2019 than pathways either involved in degradation, nutrient utilization, and assimilation or the more temporally conserved pathways involved in the generation of precursor metabolites and energy. Pathways showing the highest variation in their relative abundance were involved in the biosynthesis of tetrapyrroles, metabinding cofactors that contribute to the shell pigmentation of mollusks (reviewed within [48]), including the Pacific oyster, *Crassostrea gigas* [49]. Mollusk shell colors can vary over time, depending on changing substrate, life stage, abiotic factors including temperature and salinity, and in many species’ diets [48]. The second most variable inferred series of pathways involved the synthesis of menaquinones, lipid molecules that are important for not only the process of molluscan shell development [50] but also marine mussel adhesion to underwater surfaces [51]. The biosynthesis of secondary molecules, including enterobactins, not synthesized by mussels but found in marine mussel foot adhesive proteins, were also variable across species [52], although their potential role in freshwater mussels is unknown. These data indicate that not only is the gastrointestinal microbiome important for the diet of the host mussel species as previously suggested [11,12], but it may also be important for the physical development of the host and its activity [17].

It is currently unknown how the potential bacterial functionality and relative stability may correlate to the health or metabolic pathways of the mussel holobiont, and the future incorporation of additional data, specifically metabolomic data, would be beneficial [53]. While the metabolic pathways within wild, captive, and relocated populations of *Amblema plicata* (Unionidae) are known to significantly vary for up to one year post disturbance [54,55], how this correlates to the structure and functionality of their microbial populations has not yet been unexplored. Further research into the functional role of the bacteria within the holobiont, the correlation of the gut communities with host health, and the contribution of the holobiont to ecosystem services is highly recommended, especially utilizing transcriptomic, metabolomic, and proteomic techniques. The data from this study when combined with additional omics data could support the creation of ecological biomarkers, not only to assess the health of vulnerable mussel populations but also to determine the stability and resilience of riverine ecosystems [53].

Finally, we detected no pattern of taxonomic, phylogenetic, or functional phylosymbiosis, similar to previous analyses on freshwater mussels [12]. The lack of phylosymbiosis was consistent with our prior study, even with the greater number of individuals per species collected in this study. The continued absence of a pattern again suggests that the composition of the species present [56], or the number of species present [57], may be masking the signal or that there is a true lack of a phylosymbiosis. It is important to ensure that a significantly positive phylosymbiosis pattern is not temporally restricted, so sampling over multiple years should be an important consideration in phylosymbiosis studies. This study further suggests that factors other than host phylogenetic history are important in shaping the structure of the gut microbiome within freshwater mussels.

If environmental parameters are important factors in maintaining the gut microbiome of freshwater mussels, then anthropogenic disturbances to riverine ecosystems may not only destabilize their gut microbial communities (dysbiosis) but also detrimentally impact holobiont diet, development, and activity. Our current and previous [12] findings suggest that the temporal and geographical stability of the gastrointestinal microbiome of freshwater mussels, in combination with various host traits, correlates to the resilience of the holobiont to environmental perturbation. By understanding how the structure of microbial communities within endangered and nonthreatened mussel species respond to environmental parameters, we can begin to create biological markers to protect imperiled mussel populations and riverine ecosystems.

## Figures and Tables

**Table 1:** R^2^ values for significant pairwise temporal comparisons of alpha indices and beta dissimilarity indices of six species of Unionidae mussels sampled at the same site on the Sipsey River, AL, USA, across two years. Only significant comparisons (*p* < 0.05) are shown. Each species is represented by 3–5 individuals in 2016 and 8–10 individuals in 2019.

Tribe	Species	S(obs)	Shannon	Inverse Simpson	Bray Taxonomic	Uni-Frac Phylogenetic	Bray Functional
Lampsilini	*Lampsilis ornata*	-	-	-	0.201	0.198	0.315
	*Obovaria unicolor*	-	-	-	0.254	0.296	-
Pleurobemini	*Elliptio arca*	-	-	-	0.243	0.253	-
	*Fusconaia cerina*	-	-	-	0.250	0.223	0.417
Quadrulini	*Cyclonaias asperata*	-	-	-	0.230	0.220	-
	*Tritogonia verrucosa*	-	-	-	-	-	-

## Data Availability

Raw sequences are deposited in the NCBI Sequence Reads Archive under the BioProject ID PRJNA668192.

## Supplementary Materials

The following are available online at https://www.mdpi.com/2076-2607/9/2/411/s1, Figure S1: Boxplots representing the distribution of OTU richness and diversity (Simpson indices, expressed in an effective number of species) of six species of Unionidae mussels sampled at the same site on the Sipsey River, AL, USA, across two years. Table S1: Number of individuals collected from six species of Unionidae mussels sampled at the same site on the Sipsey River, AL, USA, across two years. Table S2 Physicochemical data at the same site on the Sipsey River, AL, USA, across two years. Table S3 Genbank identifiers for sequences that were used to construct the mussel phylogeny for the six species of mussels sampled from two sites in the Sipsey River, AL, USA. Table S4 Identity and network parameters of strong (rho >0.75) significant (adjp < 0.05) co-occurring bacterial phyla identified in the microbiomes of six species of Unionidae mussels sampled at the same site on the Sipsey River, AL, USA, across two years.
