# Supplementary material for: Gut Microbiomes of Freshwater Mussels (Unionidae) Are Taxonomically and Phylogenetically Variable across Years but Remain Functionally Stable
Source: Microorganisms. 2021 Feb 16;9(2):411. doi: 10.3390/microorganisms9020411 (PMC7920476; doi:10.3390/microorganisms9020411)
Supplement: Supplementary file 1 [file microorganisms-09-00411-s001.pdf]

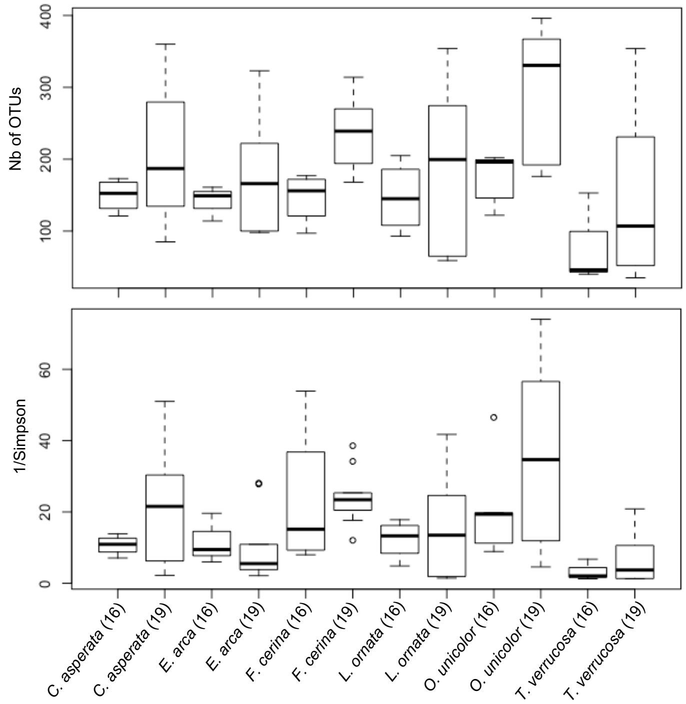

Supplementary Figure 1: Boxplots representing the distribution of OTU richness and diversity (Simpson indices, expressed in effective number of species) of six species of Unionidae mussels sampled at the same site on the Sipsey River, AL, USA, across two years. Overall differences between samplings were assessed using a Kruskall-Wallis test, with no significant differences detected. Mussel species were *Cyclonaias asperata, Elliptio arca, Fusconaia cerina, Lampsilis ornata, Obovaria unicolor* and *Tritogonia verrucosa*.

Supplementary Table 1: Number of individuals collected from six species of Unionidae mussels sampled at the same site on the Sipsey River, AL, USA, across two years. "First reported" column refers to the study were the sequences for the microbiome for a given species were originally published. The phylogenetic tribe of each species (a subfamily-level classification commonly used in freshwater mussels) is indicated, according to Lopes-Lima et al. 2016. The age-length regressions were performed using data published by Haag & Rypel, 2011.

Tribe	Mussel species	Samples (2016)	First reported	Average Size (cm)	Predicted Age (y)	Samples (2019)	First reported	Average Size (cm)	Predicted Age (y)
Lampsilini	*Lampsilis ornata*	4	Weingarten et al., 2019	84.5±15.4	8.9±0.7	9	this study	82.5±13.3	8.9±0.4
	Obovaria unicolor	5	Weingarten et al., 2019	37.6±7.0	16.9±1.4	10	this study	32.4±5.15	16.2±1.1
Pleurobemini	*Elliptio arca*	4	McCauley et al., 2020	64.3±10.1	12.6±0.6	10	this study	60.9±15.1	12.3±1.2
	Fusconaia cerina	4	Weingarten et al., 2019	46.3±6.1	17.2±0.8	10	this study	40.6±12.3	15.4±1.7
Quadrulini	*Cyclonaias asperata*	4	Weingarten et al., 2019	48.6±4.8	18.9±0.7	10	this study	43.3±7.31	18.1±1.1
	Tritogonia verrucosa	4	McCauley et al., 2020	84.6±12.1	24.5±0.9	10	this study	78.5±15.3	23.4±2.1

Supplementary Table 2: Physicochemical data at the same site on the Sipsey River, AL, USA, across two years. Three measurements of each environmental parameter were recorded between in July 2016 and June 2019. Data shown reflects the means of these three measurements and the standard error.

Year	Temperature (ºC)	pH	DO (mg/L)	DO (%)	Ammonia (ug/L)	Orthophosphate (ug/L)	Nitrate/Nitrite (ug/L)	Discharge (m^3/sec)
2016	27.5 ± 0.5	7.24 ± 0.1	6.96 ± 0.1	88.1 ± 0.5	8.4 ± 1.3	2.2 ± 1.1	179.1 ± 1.8	0.24 ± 0.1
2019	26.8 ± 0.6	7.17 ± 0.1	7.1 ± 0.1	87.5 ± 0.5	10.3 ± 0.6	2.4 ± 0.5	183 ± 2.8	0.24 ± 0.1

Supplementary Table 3: Genbank identifiers for sequences that were used to construct the mussel phylogeny for the six species of mussels sampled from two sites in the Sipsey River, AL, USA. Sequences included two nuclear markers (ITS 1, and 5.8S rRNA) and three mitochondrial markers (CO1, NADH, and 16S rRNA). Sequences of *Margaritifera margaritifera* was included as an outgroup.

Species	ITS1	5.8S	NADH	CO1	16S
Lampsilis ornata	AY365193.1	AY365193.1	AY158748.1	AY365193.1	AY365193.1
Obovaria unicolor	MH167930.1	MH167930.1	KF035419.1	KF035279.1	AF232786.1
Elliptio arca	DQ383437.1	-	AY655093.1	AY654995.1	-
Fusconaia cerina	DQ383441.1	MH133825.1	AY613792.1	DQ298538.1	AY655039.1
Cyclonaias asperata	-	-	AY158806.1	AYQ97026	-
Tritogonia verrucosa	MH362610.1	MH362610.1	MH633591.1	KT285657	AY655079.1
Margaritifera margaritifera	AY346467.1	DQ060193.1	EF446105.1	AF303342.1	DQ060167.1

Supplemental Table 4. Identity and network parameters of strong (rho >0.75) significant (adjp<0.05) co-occurring bacterial phyla identified in the microbiomes of six species of Unionidae mussels sampled at the same site on the Sipsey River, AL, USA, across two years. Bacterial phyla are measured by three topological centralities including eccentricity (shortest path length between this node and any other), degree (number of undirected edges) and stress (number of shortest paths connecting to this node) values. Species were *Lampsilis ornata*, *Obovaria unicolor*, *Elliptio arca*, *Fusconaia cerina*, *Cyclonaias asperata* and *Tritogonia verrucosa*.

Species	Year	Bacterial Phyla	Eccentricity	Degree	Stress
All Species	2016	BRC1	1	1	0
		Spirochaetes	1	1	0
		Deinococcus-Thermus	1	1	0
		Armatimonadetes	1	1	0
All Species	2019	Armatimonadetes	1	1	0
		Ignavibacteriae	1	1	0
		Proteobacteria	1	1	0
		Planctomycetes	1	1	0
L. ornata	2016	Fusobacteria	1	1	0
		Cyanobacteria	1	1	0
	2019	Armatimonadetes	1	1	0
		Ignavibacteriae	1	1	0
		Bacteroidetes	1	1	0
		Chloroflexi	1	1	0
		Proteobacteria	1	1	0
		Planctomycetes	1	1	0
O. unicolor	2016	Deinococcus-Thermus	1	1	0
		Armatimonadetes	1	1	0
	2019	Armatimonadetes	1	1	0
		Ignavibacteriae	1	1	0
		Bacteroidetes	1	1	0
		Chloroflexi	1	1	0
		Proteobacteria	1	1	0
		Planctomycetes	1	1	0
E. arca	2016	Verrucomicrobia	1	1	0
		Bacteroidetes	1	1	0
	2019	Chlamydiae	1	1	0
		Planctomycetes	2	1	0
		Proteobacteria	2	1	0
		Verrucomicrobia	1	2	2
		candidate_division_WPS-1	1	1	0
F. cerina	2016	BRC1	1	1	0
		Spirochaetes	1	1	0
		Planctomycetes	1	1	0
		Fusobacteria	1	1	0
	2019	Proteobacteria	1	1	0
		Cyanobacteria	1	1	0
		Planctomycetes	1	1	0
		Actinobacteria	1	1	0
C. asperata	2016	Chloroflexi	1	1	0
		candidate_division_WPS-1	1	1	0
		Deinococcus-Thermus	1	1	0
		Acidobacteria	1	1	0
		Cyanobacteria	1	1	0
		Bacteroidetes	1	1	0
		Planctomycetes	1	1	0
		Verrucomicrobia	1	1	0
	2019	Acidobacteria	1	2	2
		Chlamydiae	2	1	0
		Chloroflexi	2	1	0
		Verrucomicrobia	1	1	0
		Cyanobacteria/Chloroplast	1	1	0
		Planctomycetes	1	1	0
		Actinobacteria	1	1	0
T. verrucosa	2016	Actinobacteria	1	1	0
		Cyanobacteria	1	1	0
	2019	Armatimonadetes	1	1	0
		Ignavibacteria	1	1	0
		Verrucomicrobia	1	2	0
		Actinobacteria	1	2	0
		Chloroflexi	1	2	0
		Chlamydiae	1	1	0
		candidate_division_WPS-1	1	1	0
